# Supplementary material for: The nature and nurture of network evolution
Source: Nat Commun. 2023 Nov 3;14:7031. doi: 10.1038/s41467-023-42856-5 (PMC10622530; doi:10.1038/s41467-023-42856-5)
Supplement: Supplementary file 1 — Supplementary Information [file 41467_2023_42856_MOESM1_ESM.pdf]

Supplementary Information:
The nature and nurture of network evolution

Bin Zhou[1], Petter Holme[2,3], Zaiwu Gong[1], Choujun Zhan[4], Yao Huang[5], Xin Lu[6], and Xiangyi Meng[7,8,*]

[1]*Collaborative Innovation Center on Forecast and Evaluation of Meteorological Disasters, the Research Institute for Risk Governance and Emergency Decision-Making, School of Management Science and Engineering, Nanjing University of Information Science and Technology, 210044, Nanjing, Jiangsu, China*
[2]*Department of Computer Science, Aalto University, FI-00076 Aalto, Finland*
[3]*Center for Computational Social Science, Kobe University, Kobe, Hyogo, 657-8501, Japan*
[4]*School of Computer, South China Normal University, 510631, Guangzhou, Guangdong, China*
[5]*School of Electrical and Computer Engineering, Nanfang College Guangzhou, 510970, Guangzhou, Guangdong, China*
[6]*College of Systems Engineering, National University of Defense Technology, 410073, Changsha, Hunan, China*
[7]*Network Science Institute and Department of Physics, Northeastern University, Boston, MA, 02115, USA*
[8]*Department of Physics, Northwestern University, Evanston, IL, 60208, USA*

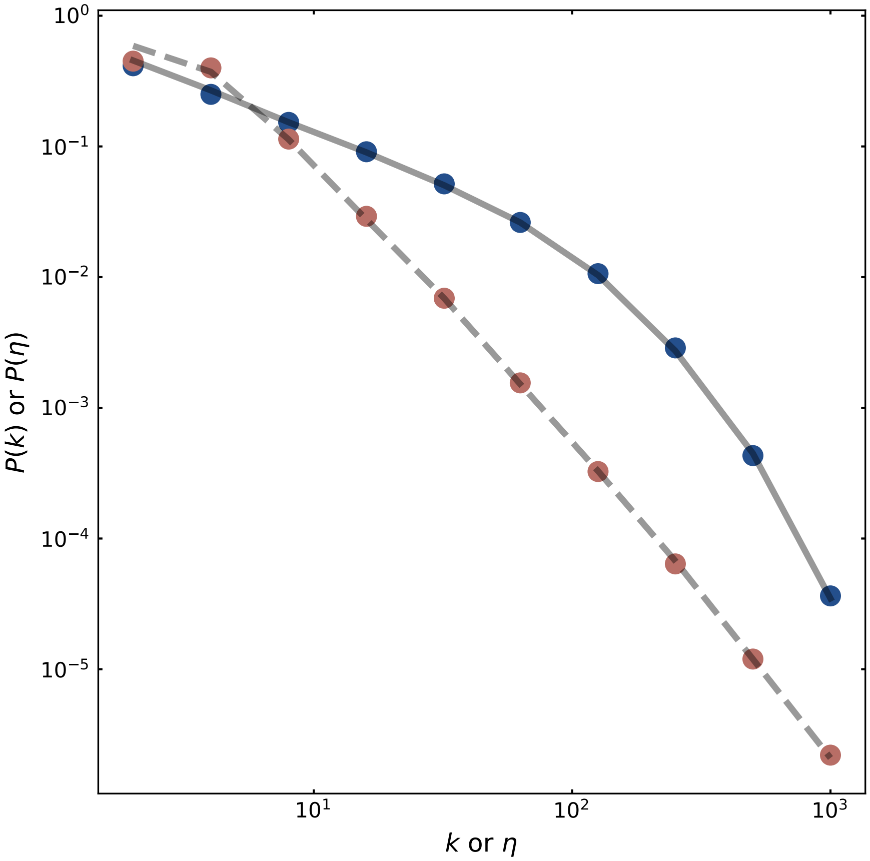

Supplementary Figure 1: Degree distribution and degree ratio distribution of the nature-nurture model. The blue and red dots represent simulation results for the degree distribution and degree ratio distribution, respectively, and the solid and dashed lines represent the analytical results [Eqs. (21) and (22) in the main text]. The parameters are chosen as $N = 1 \times 10^6$, $T = 2 \times 10^7$, $\omega_{\max} = 2$, $\alpha = 5$, and $b = 1$.

Supplementary Table 1: Descriptions of the thirty-two real-world networks.

Name	Number of nodes	Number of links	Directionality	Domain	Node interpretation	Link interpretation
01 Facebook	984, 827	92, 522, 017	Undirected	Social	User	Friendship
02 Amazon	334, 863	925, 872	Undirected	Social	User	Relationship
03 Friendster	65, 608, 365	1, 806, 067, 135	Undirected	Social	User	Friendship
04 Twitter	52, 579, 682	1, 963, 263, 821	Directed	Social	User	Following
05 Zhihu	10, 156, 825	200, 512, 859	Undirected	Social	User	Following
06 YouTube	3, 223, 643	18, 524, 095	Undirected	Social	User	Friendship
07 Weibo	7, 877, 942	700, 434, 403	Directed	Social	User	Following
08 Orkut	3, 072, 441	223, 534, 301	Undirected	Social	User	Friendship
09 LiveJournal	5, 204, 176	77, 402, 652	Undirected	Social	User	Friendship
10 MySpace	854, 498	6, 489, 736	Undirected	Social	User	Relationship
11 Telephone	233, 710	321, 562	Undirected	Social	User	Communication
12 Bitcoin	6, 297, 539	16, 057, 711	Directed	Economic	Public Key Addresses	Transactions
13 Cell	2, 239	6, 452	Directed	Biological	Protein	Interaction
14 Arabidopsis	4, 866	11, 374	Undirected	Biological	Protein	Binding
15 Linux	30, 837	213, 954	Directed	Technological	File	Inclusion
16 Internet	22, 963	48, 436	Undirected	Technological	Autonomous System	BGP Traffic
17 Patent	3, 774, 768	16, 518, 948	Directed	Technological	Patent	Citation
18 JavaScript	154, 744	517, 098	Directed	Technological	Software Package	Dependency
19 Wikipedia	2, 212, 682	24, 440, 926	Directed	Informational	Article	Hyperlink
20 Google	875, 713	5, 105, 039	Directed	Informational	Webpage	Hyperlink
21 Academia	113, 140, 646	1, 642, 237, 196	Directed	Informational	Paper	Citation
22 Yahoo	653, 260	2, 931, 708	Directed	Informational	Word	Adjacency
23 ArXiv	34, 546	421, 578	Directed	Informational	Paper	Citation
24 Baidu	2141, 300	17, 794, 839	Directed	Informational	Webpage	Hyperlink
25 BerkStan	685, 230	7, 600, 595	Directed	Informational	Webpage	Hyperlink
26 English	7, 381	46, 281	Directed	Informational	Word	Word Adjacency
27 Darkweb	7, 178	2, 5104	Directed	Informational	Domain	Hyperlink
28 DBLP	12, 590	49, 759	Directed	Informational	Paper	Citation
29 French	8, 325	24, 295	Directed	Informational	Word	Word Adjacency
30 Scientometrics	6, 571	25, 569	Undirected	Informational	Author	Collaboration
31 Spanish	11, 586	45, 129	Directed	Informational	Word	Word Adjacency
32 WordNet	146, 005	656, 999	Undirected	Informational	Word	Relationship

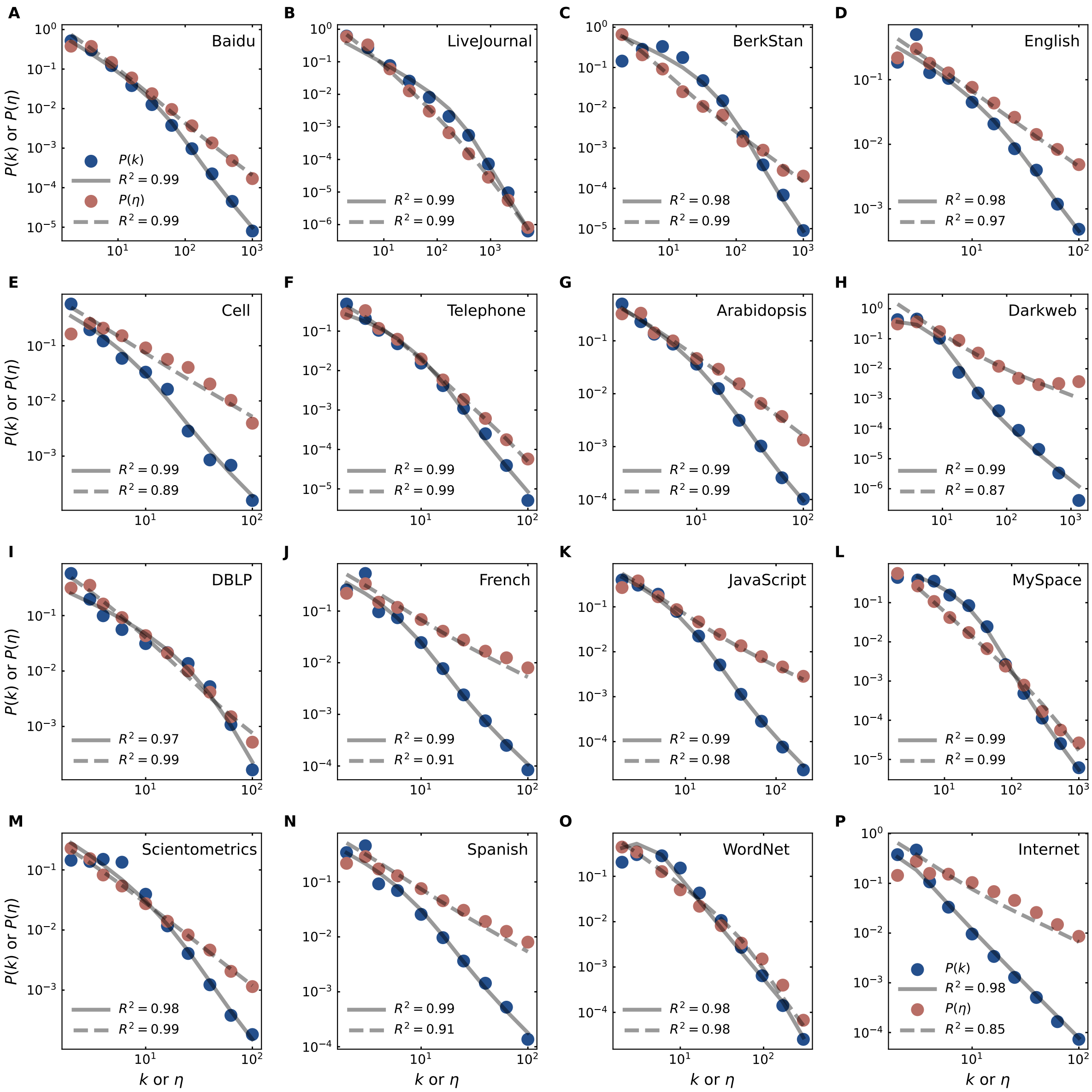

Supplementary Figure 2: Nature-nurture model fitting of real-world networks. (a-p) The observed degree distribution $P(k)$ (blue) and degree-ratio distribution $P(\eta)$ (red) in thirty-two real-world networks (other sixteen in Fig. 2 of the main text). The parameters N and T match the number of nodes and links in the empirical data. The other fitting parameters, $\omega_{\max}$, α, and b are provided in Supplementary Table 2.

Supplementary Table 2: Optimal fitting parameters with 95% confidence intervals of the nature-nurture model.

	Nature-nurture model		
	Eq. (2) and Eq. (3)		
	α	$\omega_{\max}$	b
01 Facebook	19.6 ± 3.3	2.14 ± 0.29	0.83 ± 0.26
02 Amazon	6.3 ± 1.1	3.1 ± 0.8	1.0 ± 0.4
03 Friendster	9.0 ± 4.0	1.50 ± 0.15	0.16 ± 0.05
04 Twitter	13.1 ± 1.6	2.9 ± 0.4	0.080 ± 0.029
05 Zhihu	17.9 ± 1.2	4.61 ± 0.14	0.38 ± 0.06
06 YouTube	5.5 ± 0.8	5.0 ± 0.9	0.44 ± 0.16
07 Weibo	9.0 ± 1.0	3.9 ± 0.8	1.9 ± 0.4
08 Orkut	13.2 ± 1.0	2.62 ± 0.20	1.4 ± 0.4
09 LiveJournal	12.0 ± 2.0	2.08 ± 0.14	0.13 ± 0.04
10 MySpace	7.6 ± 0.9	3.25 ± 0.30	0.59 ± 0.17
11 Telephone	6.4 ± 1.0	3.9 ± 0.7	0.62 ± 0.18
12 Bitcoin	5.4 ± 1.0	10.3 ± 3.2	0.10 ± 0.05
13 Cell	4.2 ± 1.6	6.2 ± 2.5	0.23 ± 0.12
14 Arabidopsis	5.97 ± 0.18	11.4 ± 0.8	0.0032 ± 0.0005
15 Linux	19.15 ± 0.05	17.67 ± 0.35	1.18 ± 0.05
16 Internet	2.7 ± 0.8	20.0 ± 10.0	0.85 ± 0.06
17 Patent	17.2 ± 0.5	6.2 ± 0.5	0.407 ± 0.027
18 JavaScript	5.4 ± 0.7	7.2 ± 1.0	0.31 ± 0.08
19 Wikipedia	10.1 ± 0.8	6.2 ± 0.4	0.0024 ± 0.0006
20 Google	10.16 ± 0.33	8.67 ± 0.33	0.0037 ± 0.0004
21 Academia	10.8 ± 0.8	8.4 ± 1.1	0.00015 ± 0.00009
22 Yahoo	5.91 ± 0.25	9.7 ± 0.6	0.35 ± 0.04
23 ArXiv	18.2 ± 3.0	5.0 ± 0.6	0.49 ± 0.11
24 Baidu	7.1 ± 0.6	5.4 ± 0.4	0.0118 ± 0.0023
25 BerkStan	9.3 ± 2.0	3.5 ± 0.9	0.32 ± 0.14
26 English	5.0 ± 1.6	4.4 ± 0.8	0.20 ± 0.06
27 Darkweb	4.1 ± 0.9	12.5 ± 1.6	1.7 ± 1.0
28 DBLP	9.5 ± 0.8	6.3 ± 0.8	0.23 ± 0.06
29 French	4.2 ± 1.3	8.0 ± 3.1	0.40 ± 0.21
30 Scientometrics	5.8 ± 0.5	7.5 ± 0.9	0.030 ± 0.008
31 Spanish	4.2 ± 1.3	6.7 ± 2.4	0.34 ± 0.15
32 WordNet	10.4 ± 1.3	5.6 ± 1.5	0.26 ± 0.08

Supplementary Table 3: AICc of the optimal fitting of the nature-nurture model, the nature-only model, the nurture-only model, and the nature-nurture model with $\alpha = 0$. Each row in the table is shifted by a constant so that for the nature-nurture model it is always zero.

	Nature-nurture model	Nature-only model	Nurture-only model	Nature-nurture model with $\alpha = 0$
	AICc	AICc	AICc	AICc
01 Facebook	0.00	41.91	5.56	5.84
02 Amazon	0.00	7.79	1.24	6.27
03 Friendster	0.00	25.52	3.84	−2.20
04 Twitter	0.00	53.11	22.92	21.27
05 Zhihu	0.00	23.55	7.19	6.99
06 YouTube	0.00	12.52	43.96	41.46
07 Weibo	0.00	35.13	17.17	17.61
08 Orkut	0.00	15.66	3.40	5.15
09 LiveJournal	0.00	18.92	17.08	9.53
10 MySpace	0.00	7.24	29.41	25.97
11 Telephone	0.00	17.03	7.72	14.79
12 Bitcoin	0.00	2.44	22.46	20.41
13 Cell	0.00	−2.76	15.73	13.25
14 Arabidopsis	0.00	4.44	31.72	28.18
15 Linux	0.00	2.10	17.43	15.37
16 Internet	0.00	4.54	20.85	12.44
17 Patent	0.00	16.64	11.16	9.81
18 JavaScript	0.00	8.62	34.30	32.40
19 Wikipedia	0.00	30.86	29.24	25.44
20 Google	0.00	15.56	28.13	25.46
21 Academia	0.00	1.22	10.03	6.01
22 Yahoo	0.00	21.78	48.66	47.32
23 ArXiv	0.00	10.27	1.41	2.49
24 Baidu	0.00	10.63	29.35	24.83
25 BerkStan	0.00	5.05	21.46	19.29
26 English	0.00	1.59	18.79	15.37
27 Darkweb	0.00	12.47	20.73	19.95
28 DBLP	0.00	3.28	10.82	5.16
29 French	0.00	1.68	20.96	19.27
30 Scientometrics	0.00	3.18	29.73	25.70
31 Spanish	0.00	3.24	21.9	19.64
32 WordNet	0.00	1.38	12.45	7.68

Supplementary Discussion: the range of α

In Supplementary Table 2, the optimal fitting parameter values are the values that minimize the sum of squares of residuals between the observed and predicted values of the degree distribution plus the degree ratio distribution. For a number of networks, we have $\alpha > 10$, which seems too large to be a power-law exponent. This finding suggests that the actual weight distribution may not be power law but short-tailed (e.g., exponential).

In Supplementary Table 3, the corrected Akaike information criterion is given by

$$\mathrm{AICc} = 2m - 2\ln \hat{L} + \frac{2m^2 + 2m}{n - m - 1},$$

where m is the number of fitting parameters, n the sample size, and $\hat{L}$ the maximum value of likelihood function for the model. For comparison, here we also consider the special case $\alpha = 0$, assuming the node weights follow a uniform distribution. For our analysis, the sample size is $n = 10$, and the number of fitting parameters is $m = 3, 2, 1, 2$ for the nature-nurture model, nature-only model, nurture-only model and nature-nurture model with $\alpha = 0$, respectively. The AICc value for each network represents the average of the sum of AICc value for the degree distribution and AICc value for the degree ratio distribution of the network. Each AICc shown in this table has been shifted by a constant so that for the nature-nurture model it is always zero.

By comparing the nature-nurture model (α free) and the nature-nurture model with $\alpha = 0$ (fixed), we find that the nature-nurture model (α free) is the most favored by AICc for all thirty-two real-world networks except for one network. This indicates the necessity of the assumption that node weights in the nature-nurture model adhere to a power-law (or power-law-like) distribution, which plays a significant role in the evolution of networks.

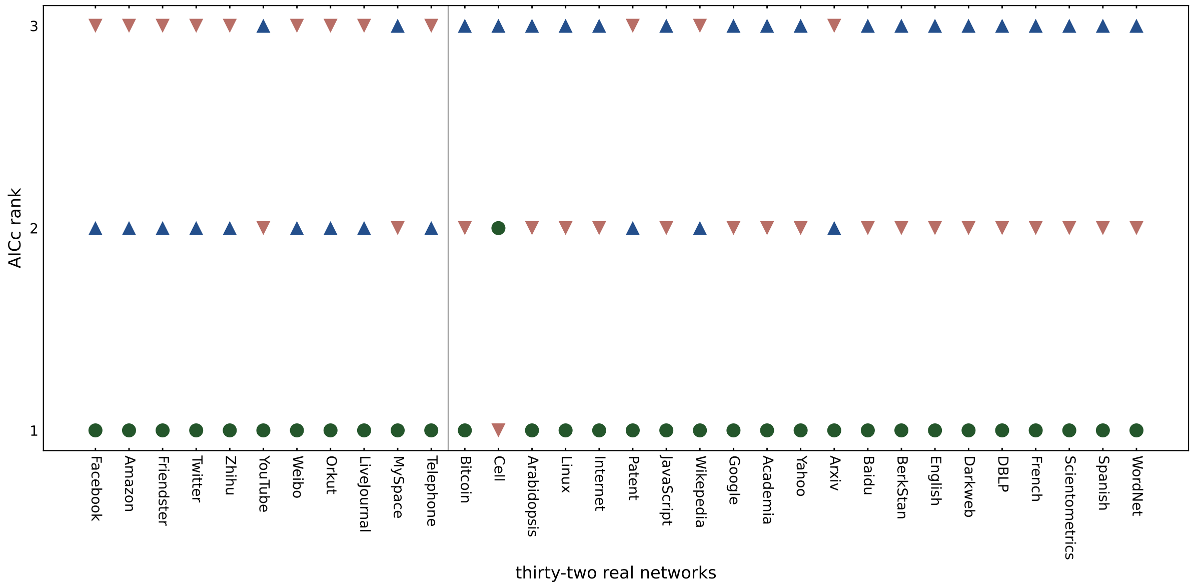

Supplementary Figure 3: Ranking the AICc for different models. AICc of the optimal fitting of the nature-nurture model (green circle), the nature-only model (red downward triangle), and the nurture-only model (blue upward triangle). For social networks (left-hand side of the vertical line), The smallest AICc values are ubiquitously obtained from the nature-nurture model (bottom), then mostly the nurture-only model (middle), then the nature-only model (top). For non-social networks (right-hand side of the vertical line), The ranking between the nurture-only model and the nature-only model is almost flipped.

Supplementary Table 4: Optimal fitting parameters of the nature-nurture model for the initial, middle, and final stages of the Academia and Zhihu networks.

	Acamedia			Zhihu		
	Initial stage	Middle stage	Final stage	Initial stage	Middle stage	Final stage
Number of nodes	$45,896,642$	$69,693,378$	$113,140,646$	$122,893$	$6,326,052$	$10,156,825$
Number of links	$300,000,000$	$820,000,000$	$1,642,237,196$	$1,000,000$	$100,000,000$	$200,512,859$
N	$45,896,642$	$69,693,378$	$113,140,646$	$122,893$	$6,326,052$	$10,156,825$
T	$300,000,000$	$820,000,000$	$1,642,237,196$	$1,000,000$	$100,000,000$	$200,512,859$
α	10.8013	10.8013	10.8013	17.8707	17.8707	17.8707
$w_{\max}$	8.3584	8.3584	8.3584	4.6088	4.6088	4.6088
b	0.0001	0.0001	0.0001	0.3783	0.3783	0.3783

Supplementary Table 5: AICc of the optimal fitting of the nature-nurture model, the nature-only model, and the nurture-only model for the initial, middle, and final stages of the Academia and Zhihu networks. Each row in the table is shifted by a constant so that for the nature-nurture model it is always zero.

	Nature-nurture model	Nature-only model	Nurture-only model
	AICc	AICc	AICc
1 Academia (initial)	0.00	13.46	24.98
2 Academia (middle)	0.00	38.25	47.14
3 Academia (final)	0.00	1.22	10.03
4 Zhihu (initial)	0.00	14.86	3.70
5 Zhihu (middle)	0.00	28.46	11.46
6 Zhihu (final)	0.00	23.55	7.19
